# DNA-release by *Streptococcus pneumoniae* autolysin LytA induced Krueppel-like factor 4 expression in macrophages

**DOI:** 10.1038/s41598-018-24152-1

**Published:** 2018-04-10

**Authors:** Toni Herta, Aritra Bhattacharyya, Christian Bollensdorf, Christin Kabus, Pedro García, Norbert Suttorp, Stefan Hippenstiel, Janine Zahlten

**Affiliations:** 1 Department of Internal Medicine/Infectious Diseases and Pulmonary Medicine, Charité - Universitätsmedizin Berlin, Augustenburger Platz 1, 13353 Berlin, Germany; 2 Departamento de Microbiología Molecular y Biología de las Infecciones, Centro de Investigaciones Biológicas, Consejo Superior de Investigaciones Científicas, Ramiro de Maeztu 9, 28040 Madrid, Spain; 3 CIBER de Enfermedades Respiratorias, Madrid, Spain

## Abstract

The recruitment of myeloid cells to the lung is of utmost importance for the elimination of invading pathogens. We investigated the *Streptococcus pneumoniae*-dependent induction mechanism of KLF4 in macrophages as a potential regulator of the macrophage immune response. We demonstrated that only viable pneumococci, which have direct contact to the host cells and release LytA-dependent DNA, induced KLF4. Exogenous supplementation of pneumococcal, other bacterial, eukaryotic foreign (human) or self (mouse) DNA to autolysis-deficient pneumococci restored (at least in part) pneumococci-related KLF4 induction. Experiments using TLR9, TRIF and MyD88 knockout macrophages revealed that TLR9, TRIF and MyD88 were partly involved in the *S. pneumoniae*-induced KLF4 expression. BMMs missing important DNA receptor related molecules (ASC^−/−^, STING^−/−^) showed no differences in pneumococci-related KLF4 expression. Similar results were observed with IFNAR^−/−^ BMMs and Type I IFN stimulated cells. LyzMcre mediated knockdown of KLF4 in BMMs resulted in a decreased secretion of proinflammatory cytokines and enhanced IL-10 release. In summary, we showed that pneumococci-related KLF4 induction in macrophages is mediated via a PAMP-DAMP induction mechanism involving a hitherto unknown host cell DNA sensor leading to a more proinflammatory macrophage phenotype.

## Introduction

The innate immune response has to be robust, rapid, and highly efficient to kill invading pathogens. During the battle between host and pathogen, human cells release numerous mediators, acting on both the invaders and the host tissue. If the host response is too weak to eliminate the pathogens, then the risk of systemic spread of bacteria with life threatening septicemia increases. On the other hand, in concert with the release of pathogen products (*e.g*. bacterial toxins like pneumococcal pneumolysin^1^) proinflammatory and toxic agents from the host’s immune response itself may impair tissue function. Thus, the innate immune response must not only be able to kill the pathogens but also has to be tightly controlled. This delicate balance is highlighted particularly in pneumonia, which is one of the major causes of death worldwide. *Streptococcus (S.) pneumoniae* is the most common source of community acquired pneumonia (CAP) across all severities^2^.

Initially, pneumococci like other bacteria are recognized by different receptors of the innate immune system, so-called pattern recognition receptors (PRRs). The PRRs include transmembrane Toll-like receptors (TLRs), cytosolic Nod-like receptors (NLRs), RIG-I-like receptors (RLRs) and various cytosolic DNA sensors^3^. Pneumococcal cell wall components such as (lipo)teichoic acids, lipoproteins and peptidoglycan fragments are recognized mainly by TLR2^4,5^ and NOD2^6^. Pneumolysin (Ply) activates the inflammasome-forming protein NLRP3^7,8^ and was described to be a ligand for TLR4^9^. Apart from TLR9 which detects unmethylated CpG motifs found in bacterial DNA^10^, there are other cytosolic DNA sensors which have been described to potentially recognize not only bacterial DNA but also DNA from foreign vertebrates or self-DNA^11^. Those potential DNA sensors include AIM2 that induces inflammasome formation, DHX9 which seems to regulate NF-κB-dependent gene expression and some others (DAI/ZBP, RNA polymerase III, LRRFIP1; IFI16, DHX36, DDX41) which control the production of type I interferons (IFN)^11,12^. For signal transduction, DNA sensors use the common adapter proteins STING (stimulator of interferon genes), ASC (apoptosis-associated speck-like protein containing a card) or MyD88 (myeloid differentiation primary response gene 88)^11,13^. Released type I IFN is recognized via the IFNα/β receptor (IFNAR), mediates the expression of IFN-regulated genes (IRGs) and inhibits the induction of proinflammatory cytokines such as KC^14,15^.

*S. pneumoniae* expresses five cell wall hydrolases. Autolysin (Lyt) A is the main autolysin and is together with LytC responsible for the release of *e.g*. Ply, bacterial DNA and RNA^16,17^. The release of these factors directly contribute to host tissue damage^18^ and leads to the PRR-related signaling in the host innate immune response. The ligand binding to PRRs and the subsequent activation of signaling cascades cause a strong chemo- and cytokine release by pneumococci-infected lung cells^5,10,19^. The consequence is, among others, a massive recruitment of PMNs to the infected lung.

Krueppel-like transcription factors (KLFs) form a subclass of about 17 zinc finger containing DNA-binding transcription factors expressed in humans and belong to the specificity protein 1 (Sp1)/KLF zinc finger binding transcription factor family^20–22^. KLF4 was first identified as a critical factor for the establishment of the skin barrier^23^ and is expressed in different cell types such as endothelial, epithelial and myeloid cells^24–27^. Recent studies indicate an important role of KLF4 for differentiation and activation of myeloid cells^24,26,28,29^. KLF4 contains an activation domain as well as a repression domain^21^ and is therefore able to induce and suppress the transcription of different genes including molecules involved in the regulation of the immune response (*e.g*. IL-6, IL-8, IL-10, MCP-1^25,27,30^). However, in macrophages the function of KLF4 is controversially discussed: Some publications described a pro-^24,28^ and others an antiinflammatory KLF4 dependent phenotype^26,31^. We previously found that pneumococcal LytA-related release of bacterial DNA and the recognition by TLR9 induced KLF4 expression in human lung epithelial cells. KLF4 expression counteracted *S. pneumoniae*-related NF-κB activation and inflammatory IL-8 secretion and induced anti-inflammatory IL-10^27,32^. In this study, we focused on the *S. pneumoniae*-dependent induction mechanism of KLF4 in macrophages and the possible influence of KLF4 on the macrophage inflammatory cytokine response during a pneumococcal infection.

## Results

### Pneumococci-induced expression of KLF4 in wt BMMs does depend on the bacterial capsule but not on phagocytosis

**Figure 1:**
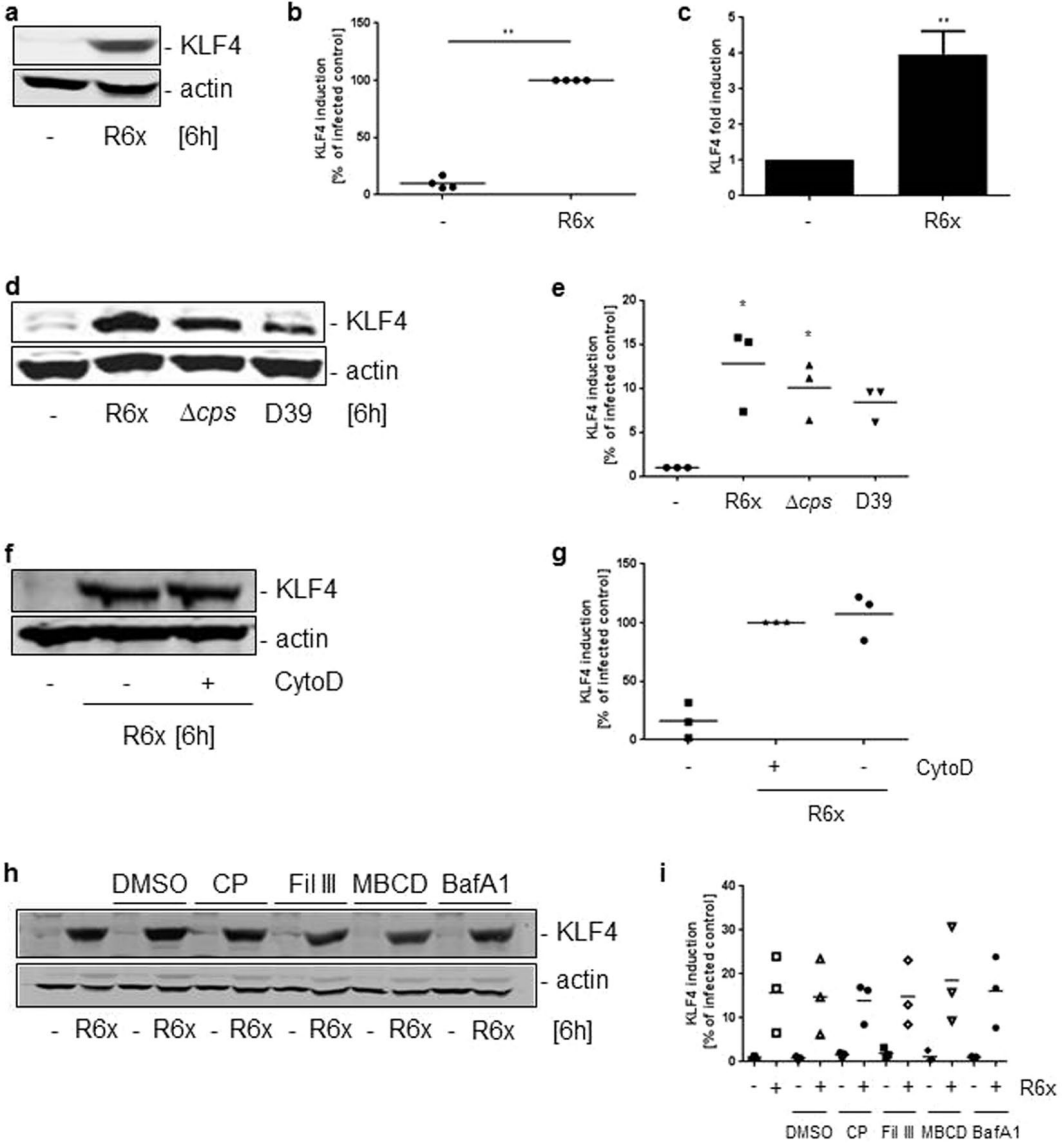
Pneumococci induce KLF4 expression in macrophages. Wild type (wt) BMMs were infected with 10^6^ CFU/ml R6x (**a**,**c**) or R6x, D39*∆cps* and D39 (10^6^ CFU/ml each) (**d**). Before stimulation with 10^6^ CFU/ml R6x wt BMMs were treated with different phagocytosis or endocytosis inhibitors dissolved in DMSO (**f**,**h**). Cytochalasin D (CytoD) (**f**), chlorpromazin hydrochloride (CP), filipin III (Fil III), L-methyl β-cyclodextrin (MBCD) and bafilomycin A1 (BafA1) (**h**). Cells were treated with DMSO only for 1 hour to rule out DMSO effects (**h**). Cell lysates were collected after 6 h and analysed for KLF4 expression and actin for equal protein load. (**a**) Shows one representative blot out of 4, (**d**), (**f**) and (**h**) out of 3 independent experiments with similar results. The densitometry of the KLF4 and actin bands of the blots was quantified using Odyssey 2.0 infrared imaging system. The ratio between the KLF4 and actin densitometry was calculated. (**b**) Shows the quantification of the blots of experiment (**a**), (**e**) for (**d**), (**g**) for (**f**) and (**i**) for (**h**). Data represents mean of 4 (**b**) or 3 (**e**,**g**,**i**) independent experiments. KLF4 RNA was quantified using qPCR (**c**). Data represents mean ± SEM of 3 independent experiments. Differences were indicated as follows: *p < 0.05; **p < 0.01.

In unstimulated wildtype bone marrow derived macrophages (wt BMMs) no expression of KLF4 was detectable (Fig. 1a–c and full-length blots Supplementary Fig. S1). Stimulation of wt BMMs for 6 h with the unencapsulated *S. pneumoniae* mutant strain R6x induced expression of KLF4 on protein (Fig. 1a,b) and RNA levels (Fig. 1c). Next, we compared KLF4 expression between the unencapsulated mutants R6x and D39∆cps and the encapsulated parent wt strain D39. We found a slightly lower KLF4 expression in wt BMMs using the encapsulated pneumococci wt strain D39 compared to the unencapsulated mutants R6x and D39∆cps (Fig. 1d,e and full-length blot Supplementary Fig. S1). The capsule of pneumococci is described, among other important functions, to prevent *S. pneumoniae* against phagocytosis^33^. To unveil a potential role of phagocytosis for the pneumococci-induced KLF4 expression we used Cytochalasin D (CytoD) to block phagocytic activity of the BMMs. Supplementary Figure S2 shows the input control of the CFU assay (supernatant plus cell lysates without gentamycin treatment) which was approximately 7.5 × 10^5^ CFU/ml. No colonies were found in the supernatants after gentamycin treatment. About 4 × 10^5^ CFU/ml phagocytosed *S. pneumoniae* were found in the cell lysates without CytoD in contrast to 0 intracellular (IC) pneumococci in the CytoD pretreated BMMs (Supplementary Fig. S2). Surprisingly, there were no differences in *S. pneumoniae*-related KLF4 expression between CytoD treated and untreated BMMs (Fig. 1f,g and full-length blot Supplementary Fig. S1). Same results were obtained using the endocytosis inhibitors filipin III (5 µg/ml), L-methyl β-cyclodextrin (10 mM) and chlorpromazine hydrochloride (14 µM) or the phagocytosis inhibitor bafilomycin A1 (25 nM) (Fig. 1h,i and full-length blot Supplementary Fig. S1).

### Pneumococci-induced KLF4 expression in BMMs is only partly mediated by TLR9, TRIF and MyD88

**Figure 2:**
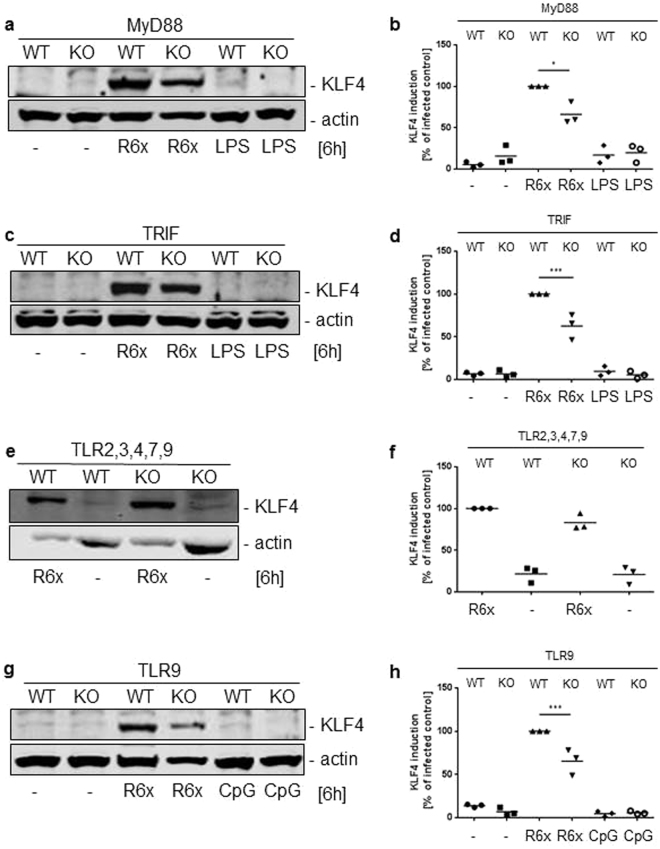
Pneumococci-induced KLF4 expression is partly mediated by TLR9, MyD88 and TRIF. MyD88- (**a**), TRIF- (**c**), TLR2,3,4,7,9- (**e**) and TLR9- (**g**) knockout BMMs were stimulated with 10^6^ CFU/ml R6x and 100 ng/ml LPS (**a**,**c**) or 1 µg/ml CpG (**g**). Cell lysates were collected after 6 h and analysed for KLF4 expression and actin for equal protein load. All blots show one representative blot out of 3 independent experiments with similar results. The densitometry of the KLF4 and actin bands of the blots was quantified using Odyssey 2.0 infrared imaging system. The ratio between the KLF4 and actin densitometry was calculated. (**b**) shows the quantification of the blots of experiment (**a**), (**d**) for (**c**), (**f**) for (**e**) and (**h**) for (**g**). Data represents mean of 3 independent experiments. Differences were indicated as follows: *p < 0.05; ***p < 0.001.

**Figure 3:**
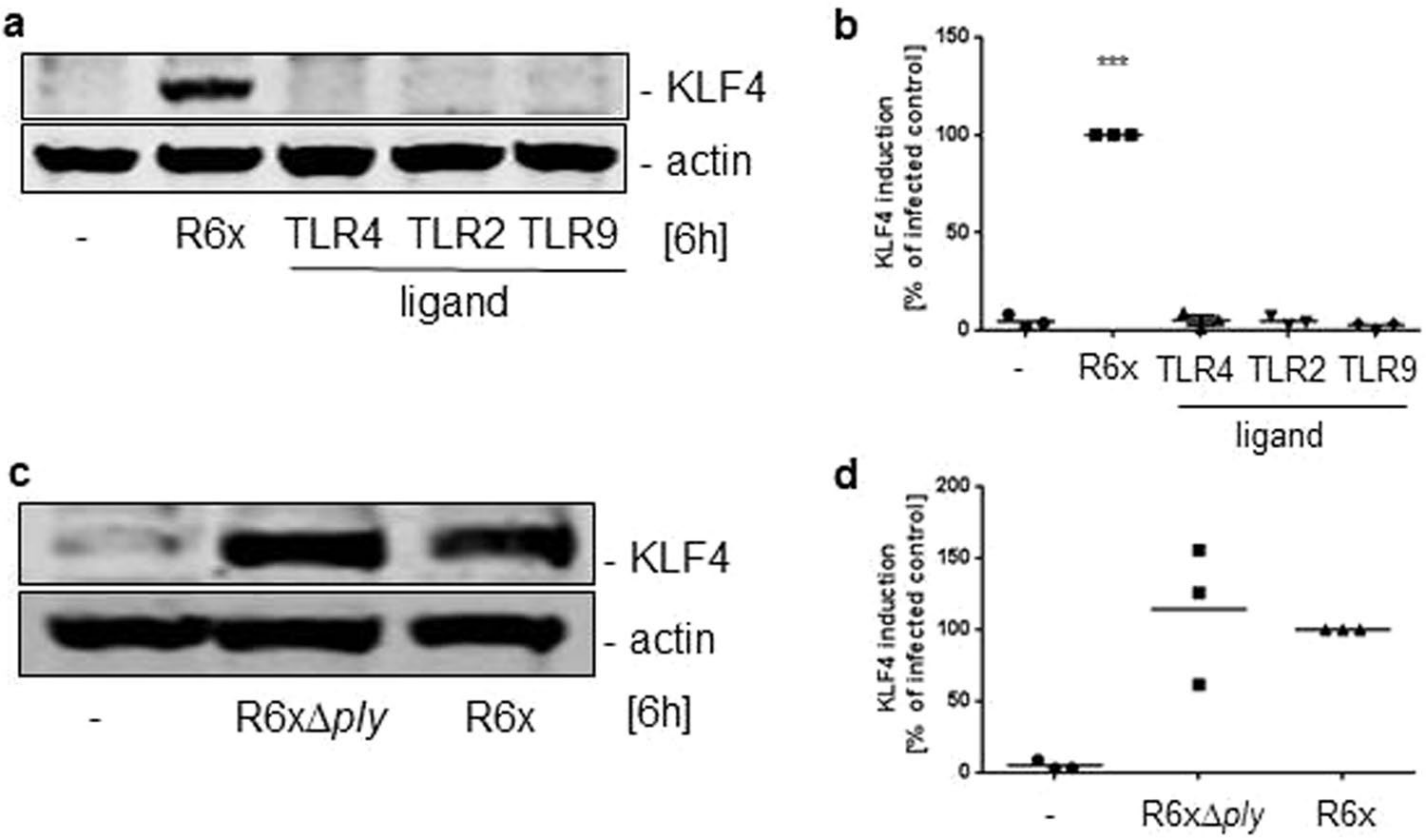
Pneumococci-induced KLF4 expression is not induced by TLR ligands or pneumolysin (ply). Wt BMMs were stimulated with 10^6^ CFU/ml R6x, 100 ng LPS (TLR4 ligand), 100 ng/ml MALP-2 (TLR2 ligand) or 1 µg/ml CpG (TLR9 ligand) (**a**) or with either 10^6^ CFU/ml R6x or 10^6^ CFU/ml R6xΔ*ply* (**c**). Cell lysates were collected after 6 h and analysed for KLF4 expression and actin for equal protein load. All blots are representatives out of 3 independent experiments with similar results. The densitometry of the KLF4 and actin bands of the blots was quantified using Odyssey 2.0 infrared imaging system. The ratio between the KLF4 and actin densitometry was calculated. (**b**) Shows the quantification of the blots of experiment (**a**) and (**d**) for (**c**). Data represents mean of 3 independent experiments. Differences were indicated as follows: ***p < 0.001.

Because we and others previously demonstrated that KLF4 expression could be induced by TLR-dependent pathways in different cell types^24,26,27,34^, we stimulated MyD88-, TRIF-, TLR2,3,4,7,9- and TLR9-deficient BMMs with 10^6^ CFU/ml R6x and LPS or CpG for 6 h. Neither TLR-related adapter protein nor TLR2,3,4,7,9-deficiency abolished *S. pneumoniae-*induced KLF4 expression (Fig. 2a–h and full-length blots Supplementary Fig. S3). In MyD88^−/−^, TRIF^−/−^ and TLR9^−/−^ BMMs the KLF4 expression seemed to be reduced (around 25% compared to wt BMM) but not completely blocked compared to the pneumococci-induced KLF4 expression in wt BMMs (Fig. 2a–d and g,h). To crosscheck these results wt BMMs were stimulated with LPS, MALP-2 or CpG (Fig. 3a,b and full-length blot Supplementary Fig. S4) solely or as a mixture of different pneumococci-related PRR ligands (PRRL mix contains LPS, MALP-2, MDP and CpG) (Supplementary Fig. S5 and full-length blot Supplementary Fig. S6). Neither the stimulation with the PRRLs alone nor the PRRLs mixture were able to induce KLF4 expression in wt BMMs. Because Ply is potentially recognized by TLR4 and the initiated response may differ from LPS/TLR4 signaling, wt BMMs were stimulated with either R6x or R6xΔ*ply* for 6 h. In line with the stimulation of wt BMMs with LPS we did not observe any differences between Ply competent and Ply-deficient pneumococci in their ability to induce KLF4 expression (Fig. 3c,d and full-length blot Supplementary Fig. S4). Since NOD2 also recognizes pneumococci, NOD2-deficient BMMs were stimulated with 10^6^ CFU/ml R6x and 100 ng/ml MDP for 6 h and analysed for KLF4. Likewise, we found no influence of NOD2 receptor-related signaling concerning *S. pneumoniae*-induced KLF4 expression (Supplementary Fig. S5 and full-length blot Supplementary Fig. S6).

### *S. pneumoniae* viability, replication, autolysis and direct contact to host cells is needed for KLF4 induction in BMMs

**Figure 4:**
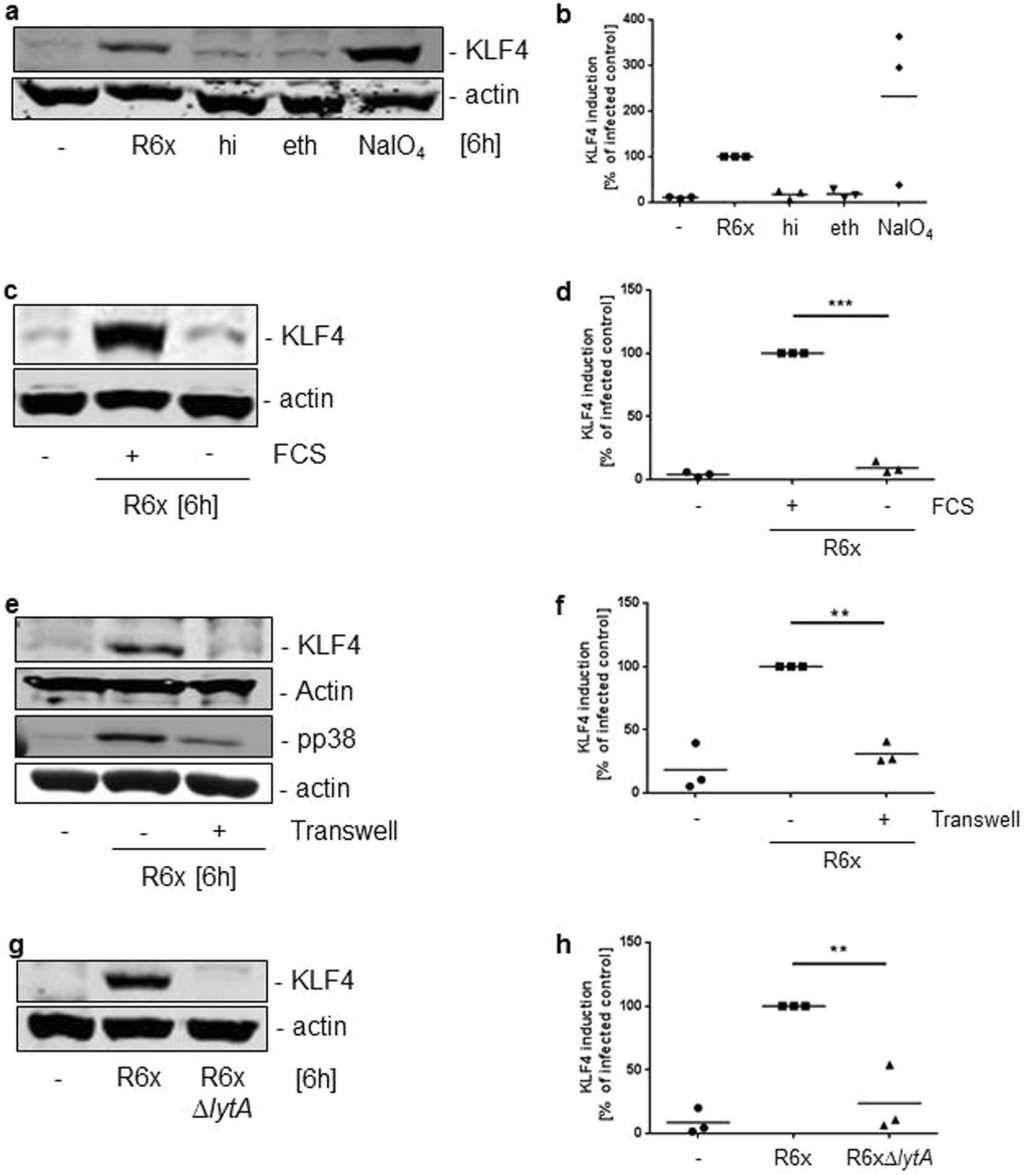
*S. pneumoniae*-dependent KLF4 expression is induced by viable, replicating bacteria which undergo autolysis and are in direct contact to the host cells. Wt BMMs were stimulated with 10^6^ CFU/ml viable (R6x and NaIO_4_-inactivated R6x) or 10^7^ CFU/ml dead (heat- (hi) and ethanol-inactivated (eth) R6x) pneumococci (**a**). The need of bacterial replication was tested by a stimulation of wt BMMs with 10^6^ CFU/ml R6x in RPMI with or without 10% FCS (**c**). Wt BMMs were stimulated with 10^6^ CFU/ml R6x in direct contact or separated using a transwell system (**e**) or with R6x and R6xΔ*lytA* (10^6^ CFU/ml each) (**g**). Cell lysates were collected 6 h after stimulation and analysed for KLF4 expression. Actin was used to confirm equal protein load. pp38 proved stimulation efficiency (**e**). All blots are representatives out of 3 independent experiments with similar results. The densitometry of the KLF4 and actin bands of the blots was quantified using Odyssey 2.0 infrared imaging system. The ratio between the KLF4 and actin densitometry was calculated. (**b**) shows the quantification of the blots of experiment (**a**), (**d**) for (**c**), (**f**) for (**e**) and (**h**) for (**g**). Data represents mean of 3 independent experiments. Differences were indicated as follows: **p < 0.01; ***p < 0.001.

To further investigate the KLF4-inducing factor of *S. pneumoniae* in BMMs, bacteria were treated differentially to destroy specific components (hi = heat inactivation or PK = Proteinase K or PE = Pronase E for protein digestion; eth = ethanol for lipid dissolution; NaIO_4_ for carbohydrate destruction). After treatment, bacteria were plated on blood agar plates to prove viability. Only heat inactivation and ethanol killed the bacteria, whereas all others were still able to form colonies. Only viable (NaIO4-, Proteinase K- and Pronase E-treated) but not dead pneumococci (ethanol- and heat-inactivated) were able to induce KLF4 expression regardless of the denaturized component (Fig. 4a,b with full-length blot Supplementary Fig. S7 and Supplementary Fig. S8 with full-length blot Supplementary Fig. S9), we further tested whether only the presence of viable bacteria was needed for KLF4 induction or if bacterial replication was required. To examine the need for replication we stimulated wt BMMs in RPMI medium with or without FCS because FCS is necessary for pneumococcal replication (Supplementary Fig. S8). Non-replicating *S. pneumoniae* were not able to induce KLF4 expression compared to wt BMMs stimulated with pneumococci in FCS supplemented RPMI (Fig. 4c,d with full-length blot Supplementary Fig. S7 and Supplementary Fig. S8 with full-length blot Supplementary Fig. S9). Next, we separated pneumococci from wt BMMs using a transwell system and analysed the BMM cell lysates for KLF4 expression 6 h after stimulation. As shown in Fig. 4e,f, separation of *S. pneumoniae* from macrophages abolished pneumococci-induced KLF4 expression while phosphorylated p38 (pp38) mitogen-activated protein kinase (MAPK) was still detectable (Fig. 4e,f and full-length blot Supplementary Fig. S7). Given that replicating pneumococci undergo autolysis we used unencapsulated pneumococci deficient for the main autolysin LytA, which completely abolished pneumococci-induced KLF4 expression (Fig. 4g,h and full-length blot Supplementary Fig. S7).

### Supplementation of extracellular DNA restored KLF4 induction by LytA-deficient pneumococci

**Figure 5:**
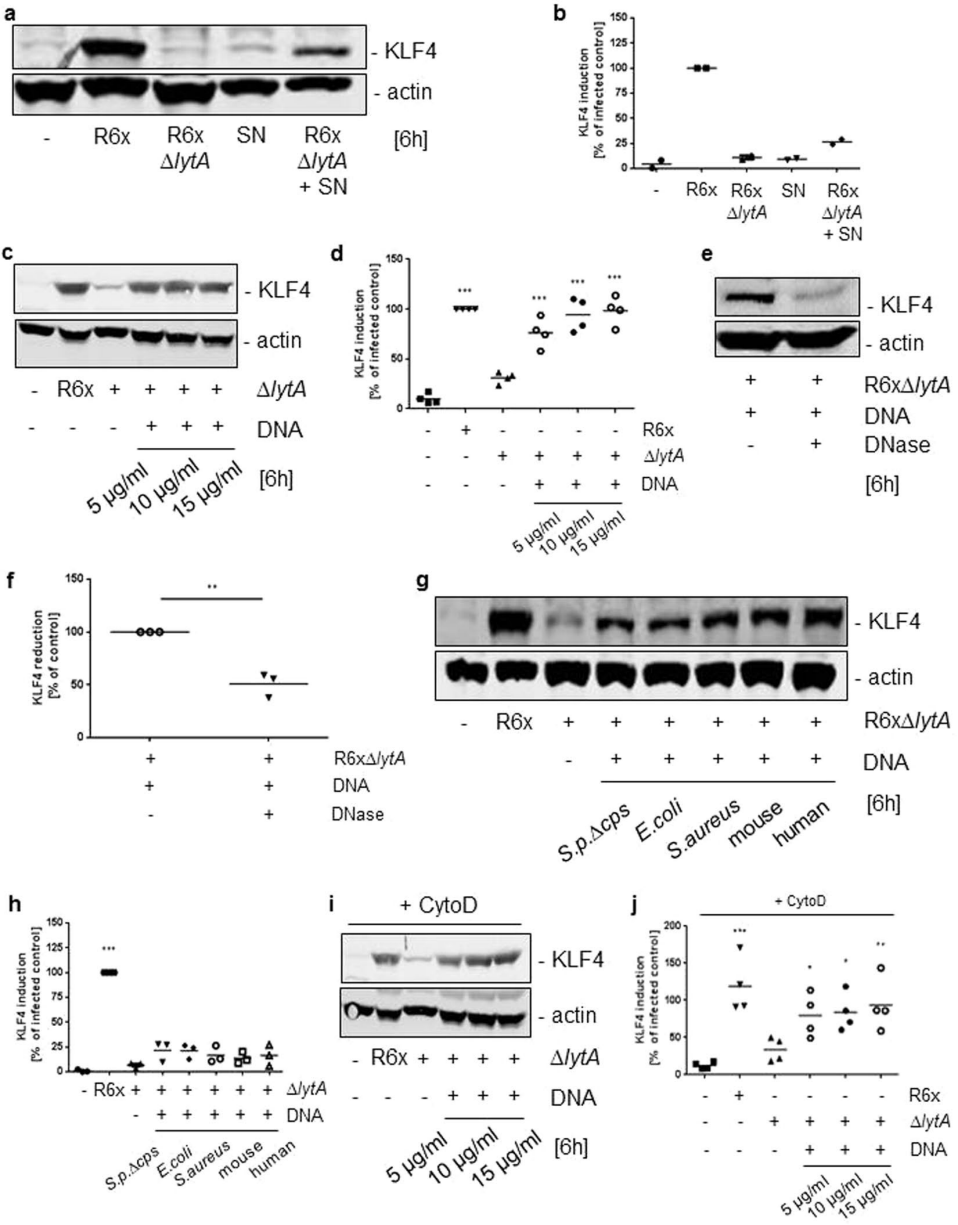
KLF4 expression is (partly) restored by addition of extracellular pro- and eukaryotic DNA to LytA-deficient S. pneumoniae mutants. Wt BMMs were stimulated with 10^6^ CFU/ml R6x, pneumococci free supernatants (SN) from *S. pneumoniae* R6x overnight cultures and 10^6^ CFU/ml R6xΔ*lytA* solely or in combination with R6x SN (**a**). Next, wt BMMs were exposed to 10^6^ CFU/ml R6x, 10^6^ CFU/ml R6xΔ*lytA* alone or in combination with 5 µg/ml, 10 µg/ml and 15 µg/ml purified *S. pneumoniae*-derived DNA (**c**). Costimulation of wt BMMs with 10^6^ CFU/ml R6xΔ*lytA* and 5 µg/ml pneumococcal DNA was repeated with or without DNase pretreatment (**e**), or in combination with purified *E. coli*, *S. aureus*, mouse- or human DNA (**g**). After Cytochalasin D (CytoD) pretreatment wt BMMs were stimulated with 10^6^ CFU/ml R6xΔ*lytA* solely or combined with 5 µg/ml, 10 µg/ml and 15 µg/ml purified *S. pneumoniae* DNA (**i**). Cell lysates were collected after 6 h and analysed for KLF4 expression. Actin was used to proof equal protein load. (**a**) Shows one representative blot out of 2, (**c**) out of 4, (**e**) and (**g**) out of 3 and (**i**) out of 4 independent experiments with similar results. The densitometry of the KLF4 and actin bands of the blots was quantified using Odyssey 2.0 infrared imaging system. The ratio between the KLF4 and actin densitometry was calculated. (**b**) shows the quantification of the blots of experiment (**a**), (**d**) for (**c**), (**h**) for (**g**) and (**j**) for (**i**). (**f**) Shows the quantified reduction of KLF4 expression after DNase pretreatment. Data represents mean of 2 (**b**), 3 (**f**,**h**) or 4 (**d**,**j**) independent experiments. Differences were indicated as follows: *p < 0.05; **p < 0.01; ***p < 0.001.

Since the LytA-deficient *S. pneumoniae* mutant strain failed to induce pneumococci-related KLF4 induction we hypothesized that the KLF4 inducing factor is released by pneumococcal autolysis. To test this hypothesis we stimulated wt BMMs with R6x-derived supernatants solely or in combination with R6xΔ*lytA*. As shown in Fig. 5a,b, neither the supernatants alone nor the stimulation with LytA-deficient pneumococci induced KLF4 expression. Noteworthy was that supplementation of pneumococcal supernatants to LytA-deficient *S. pneumoniae* restored (at least in part) KLF4 induction (Fig. 5a,b and full-length blot Supplementary Fig. S10). As one of the components released by bacterial autolysis is bacterial DNA and as it was shown in this study that bacterial DNA-recognizing TLR9 is partly involved in the KLF4 induction, we isolated pneumococcal DNA and stimulated wt BMMs with the LytA-deficient mutant strain in combination with pneumococcal DNA. Again, LytA-deficient pneumococci alone were not able to induce KLF4 expression but in combination with pneumococcal DNA KLF4 expression was restored (Fig. 5c,d and full-length blot Supplementary Fig. S10). Notably, KLF4 induction was significantly reduced when pneumococcal DNA was pretreated with DNase before stimulation (Fig. 5e,f and full-length blot Supplementary Fig. S10). Next, we stimulated wt BMMs with R6xΔ*lytA* in combination with purified DNA from other bacteria (*Escherichia* (*E*.) *coli*, *Staphylococcus* (*S*.) *aureus*) and purified eukaryotic DNA (mouse and human). As expected, KLF4 induction in wt BMMs was (at least partly) restored when costimulated with other bacterial DNA, but strikingly eukaryotic DNA was also able to induce KLF4 expression in combination with the LytA-deficient pneumococcal mutant strain (Fig. 5g,h and full-length blot Supplementary Fig. S10). In contrast, costimulation with pneumococcal RNA was not able to induce KLF4 expression in wt BMMs (Supplementary Fig. S11 and full-length blot Supplementary Fig. S12). Since TLR9 usually recognizes prokaryotic DNA, we hypothesized that one of the recently discovered cytosolic DNA sensors may participate in pneumococci-dependent KLF4 expression. Therefore, we stimulated DNA sensor-related adapter protein-deficient BMMs (ASC^−/−^ and STING^−/−^ BMMs) with R6x. However, neither ASC^−/−^ nor STING^−/−^ BMMs displayed a significant difference in KLF4 expression compared to corresponding wt BMMs (Supplementary Fig. S11 and full-length blots Supplementary Fig. S12). As induction of KLF4 does not seem to be depend on cytosolic DNA sensors we repeated the costimulation of wt BMMS with R6xΔ*lytA* and pneumococcal DNA after CytoD pretreatment to block phagocytosis. Interestingly, in line with Fig. 1f,g blocking of phagocytosis did not alter KLF4 induction (Fig. 5i,j and full-length blot Supplementary Fig. S10).

### Pneumococci-related KLF4 expression does not depend on auto-/paracrine loop of the type I IFN response

**Figure 6:**
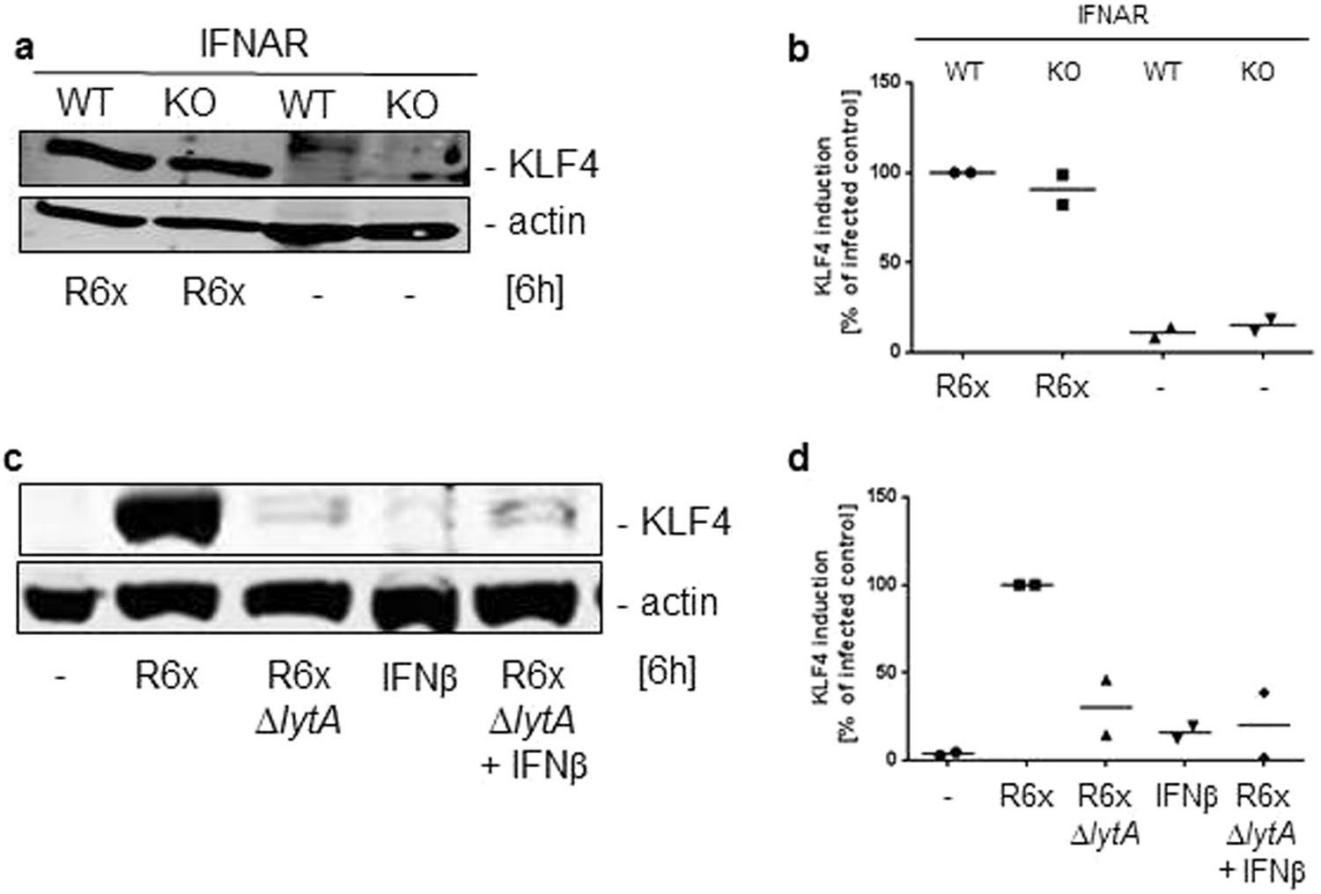
Pneumococci-dependent KLF4 induction in BMMs is not mediated by type I IFN signaling. WT or IFNAR-deficient (IFNAR KO) BMMs were stimulated with 10^6^ CFU/ml R6x (**a**) and wt BMMs were exposed to 500 U/ml IFNβ alone or in combination with 10^6^ CFU/ml R6xΔ*lytA* (**c**). Cell lysates were collected 6 h after stimulation and analysed for KLF4 expression. Actin was used to confirm equal protein load. Both blots show one representative result out of 2 independent experiments with similar results. The densitometry of the KLF4 and actin bands of the blots was quantified using Odyssey 2.0 infrared imaging system. The ratio between the KLF4 and actin densitometry was calculated. (**b**) shows the quantification of the blots of experiment (**a**) and (**d**) for (**c**). Data represents mean of 2 independent experiments.

Because activation of TLR9 and DNA sensors led to a forced type I interferon (IFN) release in macrophages^35^ we used interferon-α/β receptor (IFNAR) knockout BMMs to exclude *S. pneumoniae*-dependent KLF4 induction mediated by an auto-/paracrine type I IFN loop in wt BMMs. As shown in Fig. 6a,b the pneumococci-mediated KLF4 expression in IFNAR^−/−^ BMMs was equivalent to the wt BMMs (Fig. 6a,b and full-length blot Supplementary Fig. S13). Furthermore, stimulation with either 500 U/ml IFNβ alone or in combination with R6xΔ*lytA* was not able to induce KLF4 expression in wt BMMs (Fig. 6c,d and full-length blot Supplementary Fig. S13).

### Loss of KLF4 decreased pneumococci-induced proinflammatory cytokine release and enhanced IL-10 secretion

**Figure 7:**
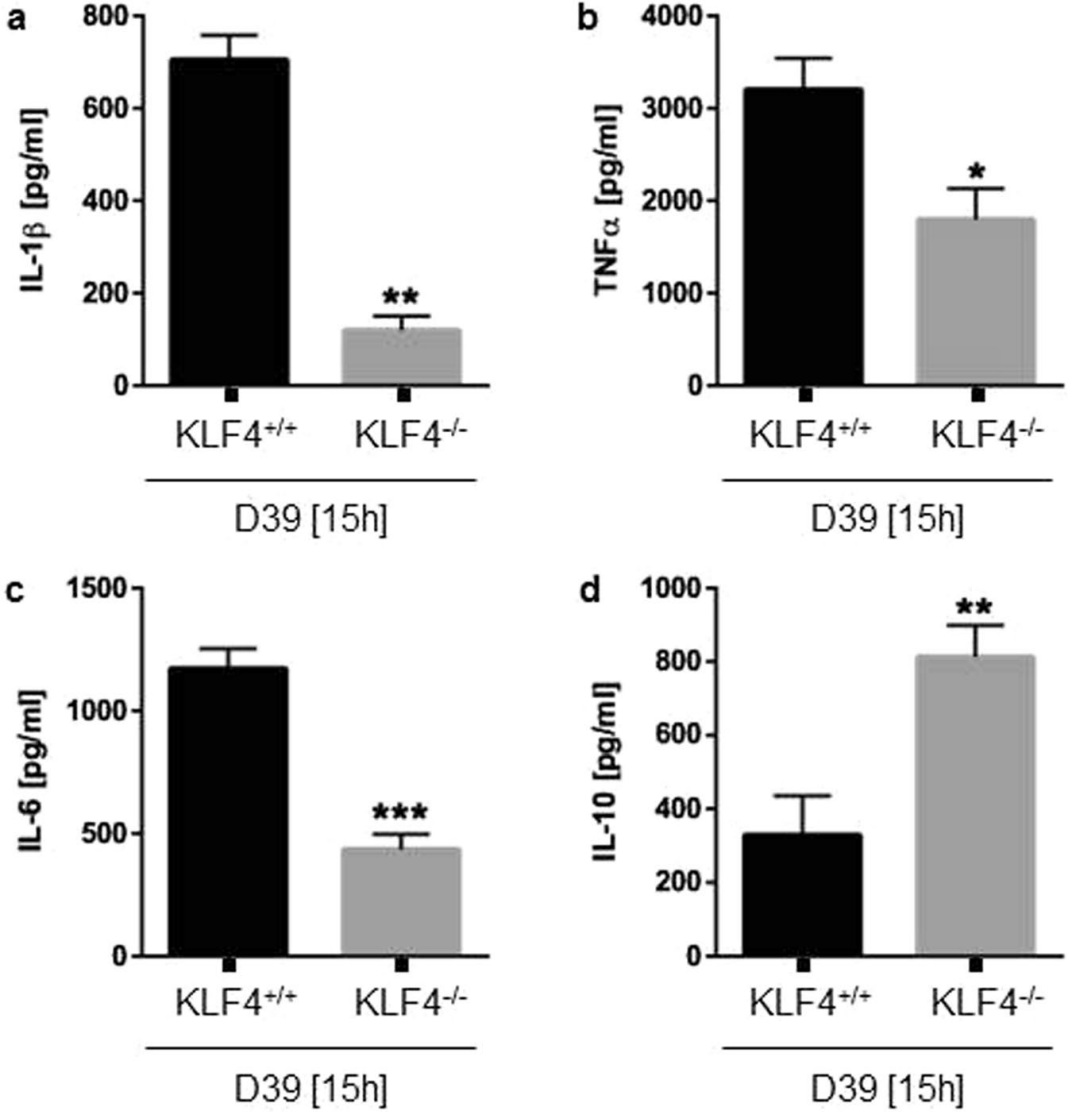
Loss of KLF4 decreases pneumococci-induced proinflammatory cytokine response and up-regulates antiinflammatory IL-10 secretion in BMMs. BMMs derived from C57BL/6 LyzMcre^−/−^/Klf4^loxP/loxP^ (KLF4^+/+^) and C57BL/6 LyzMcre^+/+^/Klf4^loxP/loxP^ (KLF4^−/−^) mice were stimulated with 10^6^ CFU/ml *S. pneumoniae* D39 for 15 h. Supernatants were collected and IL-1β (**a**), TNFα (**b**), IL-6 (**c**) and IL-10 (**d**) ELISAs were performed. Data represents mean ± SEM of 6 independent experiments. Differences were indicated as follows: *p < 0.05; **p < 0.01; ***p < 0.001.

For functional analysis of KLF4 in macrophages exposed to pneumococci we used BMMs from C57BL/6 LyzMcre^−/−^/Klf4^loxP/loxP^ (KLF4^+/+^) and C57BL/6 LyzMcre^+/+^/Klf4^loxP/loxP^ (KLF4^−/−^) mice. *S. pneumoniae*-related IL-1β (Fig. 7a), TNFα (Fig. 7b) and IL-6 (Fig. 7c) secretion was significantly reduced in KLF4^−/−^ BMMs, whereas pneumococci-related IL-10 secretion was markedly enhanced in KLF4^−/−^ cells compared to KLF4^+/+^ cells (Fig. 7d). The knockdown of KLF4 was confirmed by KLF4 western blot analysis and is shown in Supplementary Fig. S14 (full-length blot Supplementary Fig. S15).

## Discussion

Herein we investigated *S. pneumoniae*-dependent KLF4 induction in murine macrophages. Application of differently inactivated (dead or viable) *S. pneumoniae*, separation of the bacteria from the BMMs, and suppression of phagocytosis revealed that direct contact of viable bacteria to the host cells, independent of phagocytosis, is needed to induce KLF4 expression in BMMs. In a previous study, we demonstrated that pneumococci-related KLF4 expression in human bronchial-epithelial cells depended on the recognition of pneumococcal DNA by TLR9 and the related adapter molecule MyD88^27^. Experiments using TLR9^−/−^ and MyD88^−/−^ BMMs revealed that TLR9 and MyD88 are only partially involved in the pneumococci-related KLF4 expression in BMMs (~25% reduction), which already indicated a cell-specific response. Nevertheless, pneumococcal DNA seems to be an important trigger for KLF4 expression not only in bronchial-epithelial cells but also in BMMs. Exogenous supplementation of *S. pneumoniae* DNA to the *S. pneumoniae*-LytA mutant (which is unable to induce KLF4) restored KLF4 induction. The addition of DNase reduced this effect. Noteworthy was that *S. pneumoniae* DNA could be (at least partly) substituted with DNA from other bacterial species as well as with foreign eukaryotic (human) and self-DNA (mouse), but not with bacterial RNA. As TLR9 mainly detects prokaryotic DNA and only seemed to be partially involved in the induction of KLF4 we hypothesized the participation of an additional host cell DNA sensor. For this reason, we examined pneumococci-dependent KLF4 expression in BMMs missing the putative DNA receptor-related adapter proteins ASC and STING. However, we did not observe a reduction of pneumococci-related KLF4 expression. The activation of TLR9 (and other DNA sensors) induces the secretion of type I IFNs which are sensed by IFNAR in an auto-/or paracrine manner and leads to the activation of interferon regulatory factors (IRFs) which induce the expression of interferon regulated genes^35^. KLF4 was previously shown to be induced by IRF8 in monocytes^36^. Strikingly, *S. pneumoniae*-dependent KLF4 expression was neither influenced by IFNAR knockout nor by the stimulation with IFNβ. Furthermore, phagocytosis/endocytosis of DNA was not necessary to induce KLF4 although endosomal TLR9 seems to be involved. Thus, we conclude that DNA activates TLR9 in an phagocytosis-endocytosis-independent way to induce KLF4 and the putative second DNA-receptor might be located on the BMM surface (discussed later). Overall, the results suggest that the KLF4 expression is, in addition to TLR9, mediated by a hitherto unknown DNA receptor or at least by a DNA sensor-related pathway independent of phagocytosis/endocytosis, ASC, STING and type I IFNs which detects pro- and eukaryotic DNA molecules. A similar mechanism was described by Yasuda *et al*. showing a stimulation of TNFα production in peritoneal- and RAW264.7 macrophages after recognition of pro- or eukaryotic DNA by TLR9 and a not yet known second DNA-receptor^37^.

Because DNA alone was not sufficient to induce KLF4 expression, we hypothesized that at least a second signal is needed (direct contact of replicating bacteria). Using TLR2,3,4,7,9^−/−^ BMMs a reduction of KLF4 expression was seen (most likely due to TLR9^−/−^). Interestingly, TRIF^−/−^ BMMs also showed a significant reduction in KLF4 expression (~25%, similar to TLR9^−/−^ and MyD88^−/−^ BMMs), which may be explained by its recently discovered function as an adapter protein for TLR9 signaling^38^. Although TLR2, TLR4 and NOD2 are clearly implicated in the recognition of *S. pneumoniae*^6,9,19,39^, stimulation of wt BMMs with MALP-2 (TLR2-ligand), LPS (TLR4-ligand) and MDP (NOD2-ligand) alone or in a mixture of different PRR ligands including CpG DNA did not induce KLF4. Additionally, we examined the role of Ply which is an important virulence factor of *S. pneumoniae*^40–42^ that activates host cells, at least possibly in part, via recognition by TLR4^4,9^. Nevertheless, Ply-negative pneumococci still caused KLF4 expression. Our observations agree with findings from Liao *et al*. who demonstrated that KLF4 is rather down-regulated in LPS treated peritoneal macrophages or BMMs than upregulated^31^. However, there exist conflicting results from other groups who showed that KLF4 is induced by LPS in macrophages^24,26^. Given that KLF4 has a strong impact on macrophage differentiation^28,29^ and KLF4 expression in leukemia-derived monocytes may differ to monocytes obtained from healthy individuals, these inconsistent data may be caused by the use of primary macrophages (this study and^31^) versus leukemia-derived cell lines from humans and mice (THP1^24^ and RAW264.7 cells^26^). Additionally, different sources of LPS (in this study *Salmonella* (*S*.) *minnesota* versus *S. typhi*^24^ or *E*. *coli*^26^), concentrations and different exposure times may have had an impact on the variety of results from the different studies.

As we found a slightly reduced KLF4 induction using the encapsulated D39 pneumococci, this second signal may depend on pneumococcal cell wall associated components which are (at least partly) covered by the capsule. Furthermore, the host cell receptor ought to localize extracellularly because the need of phagocytosis could be excluded. Since KLF4 induction seemed not to be mediated via TLR2 or NOD2, this pneumococcal cell wall associated molecule is unlikely a peptidoglycan, lipoteichoic acid or a lipoprotein (recognized by TLR2 and/or NOD2).

**Figure 8:**
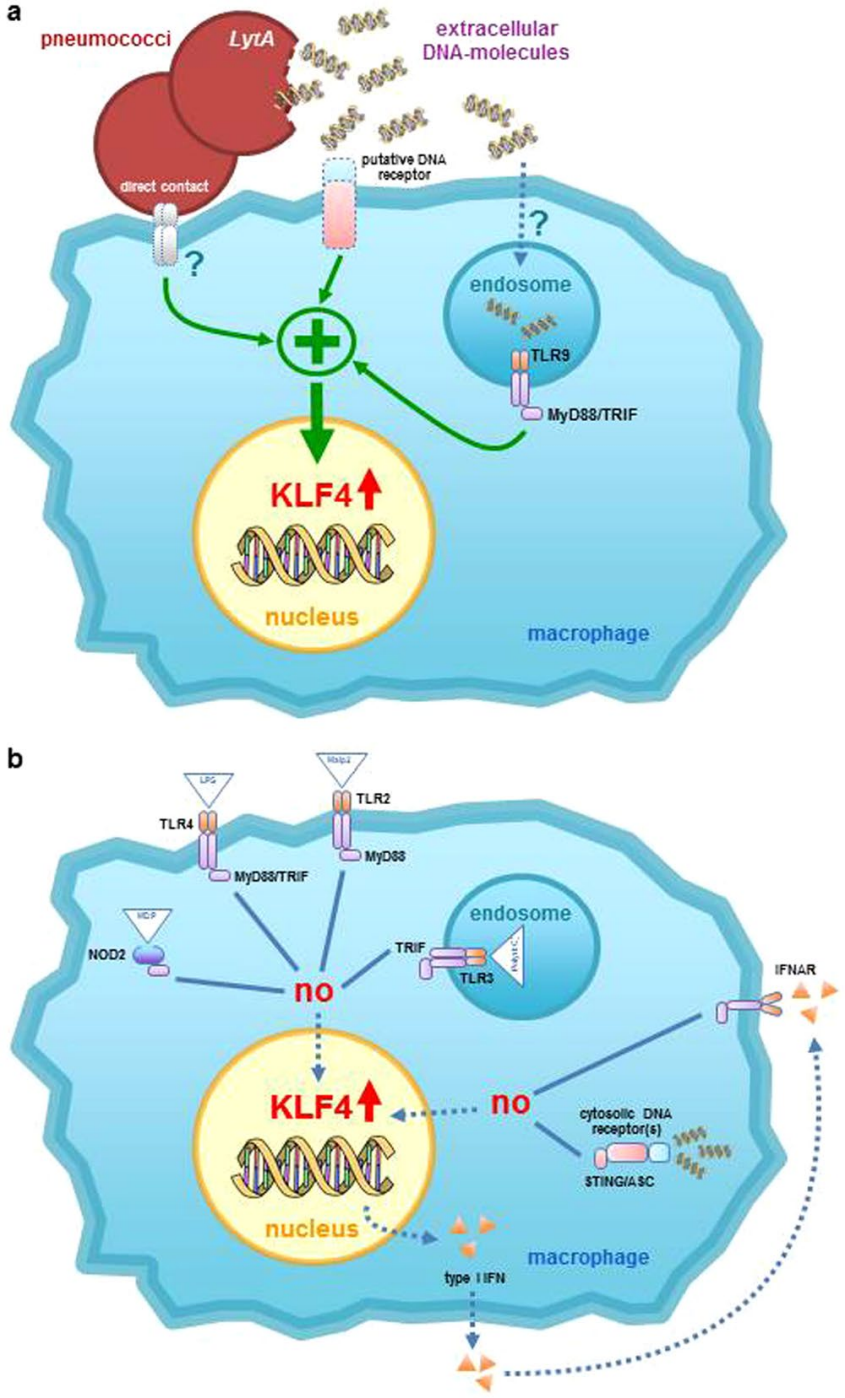
Schematic representation of identified and excluded signaling pathways. The induction of pneumococci-dependent KLF4 expression in BMMs needs direct contact of viable bacteria in combination with extracellular pro- or eukaryotic DNA. This induction is partly mediated via TLR9/MyD88/TRIF and a probably yet unknown extracellular DNA sensor (**a**). Although TLR9 seems to be partly involved in the KLF4 expression, phagocytosis is not required. We additionally could exclude the participation of the receptors TLR2, TLR4, NOD2 and IFNAR, as well as the adapter proteins STING and ASC (**b**).

As (eukaryotic) DNA is liberated by damaged tissue it can act as a DAMP (damage-associated molecular pattern) indicating danger to the host^43^. Thus, *S. pneumoniae* seems to induce KLF4 in BMMs by a PAMP-DAMP costimulatory signal (schematically summarized in Fig. 8). What could be the rationale behind this mechanism? To answer this question, we investigated the function of KLF4 in BMMs using BMMs from C57BL/6 LyzMcre^+/+^/Klf4^loxP/loxP^ mice. In these mice the knockdown of KLF4 is only induced in mature myeloid cells^44^. However, the knockdown efficiency in our experimental setting did not exceed 40% although Liao *et al*. showed 90% knockdown efficiency using a similar model^31^. In general, deletion efficiency in myeloid-Cre deleting strains shows variation influenced by factors such as sex of the parent contributing the Cre allel due to variation in Cre expression between testes and ovary^45^ which could contribute to the efficiency difference. Despite the limited KLF4 knockdown efficiency, we found a significant alteration of the pneumococci-dependent cytokine release in KLF4^−/−^ macrophages. The release of the proinflammatory cytokines IL-1β, TNFα and IL-6 was downregulated in KLF4^−/−^ BMMs compared to KLF4^+/+^ control cells while the release of the anti-inflammatory cytokine IL-10 was increased. This falls in line with observations of Feinberg and Kaushik *et al*. who postulated a proinflammatory function of KLF4 in macrophages and monocytic microglial cells^24,46^. Noteworthy was that this putative proinflammatory role of KLF4 in macrophages contrasted with results in pulmonary epithelial cells, where the loss of KLF4 enhanced pneumococci-related proinflammatory cytokines^32^ and decreased anti-inflammatory IL-10 release^27^. These findings underscore the cell-specific activation and effects of KLF4.

Assuming a proinflammatory function of KLF4 in BMMs, we speculated about the meaning of the PAMP-DAMP induction mechanism. Perhaps the two components act in a synergistic way to mount a sufficient immune response as shown for the induction of iNOS in RAW264.7 macrophages by LPS and bacterial DNA^47^. Alternatively, the second component (direct contact of replicating bacteria) possibly prevents an inadequate activation of the macrophages by DNA alone. The fact that we did not see any induction of KLF4 by one of the two components alone favours the second hypothesis. Since every immune response causes collateral damage to host tissue strict regulation is needed^48^. As we ruled out the need for phagocytosis/endocytosis, a potential DNA-sensor present on the cell membrane activated by pro- and eukaryotic DNA bears the risk of severe hyperinflammation. Perhaps direct contact of bacteria and BMMs renders this otherwise masked DNA-receptor accessible for its ligand. However, as long as the involved receptors are not clearly identified this remains speculative.

The obtained results strengthen the hypothesis that KLF4 might play a role in innate immunity in the lung. Interestingly, there seem to be strong differences between the induction and function of pneumococci-related KLF4 expression in macrophages compared to human lung epithelial cells. In this study, we showed that pneumococci-induced KLF4 expression in BMMs is mediated by a combined induction mechanism involving pro- or (in part) eukaryotic DNA and direct contact of bacteria and BMMs leading to a more proinflammatory macrophage phenotype. Whereas, in bronchial epithelial cells (as shown in previous studies) the pneumococci-dependent KLF4 induction is mediated by pneumococcal DNA alone via TLR9 and MyD88 triggering an anti-inflammatory cytokine release^27,32^. Therefore, it is tempting to speculate that KLF4 might on the one hand support the elimination of invading pneumococci by activating proinflammatory pathways in macrophages and on the other hand it might act as an inflammatory brake in the lung epithelium to prevent hyperinflammation and tissue-destruction. This hypothesis should be investigated in further studies.

## Materials and Methods

Cytochalasin D and Pronase E were purchased from Sigma-Aldrich (Seelze, Germany), ODN M362 (CpG, IFNβ and bafilomycin A1 from Invivogen (San Diego, CA, USA), MDP and MALP-2 from Alexis Biochemicals (Lörrach, Germany), LPS from *S*. *minnesota* from Enzo Life Sciences GmbH (Lörrach, Germany), Proteinase K, chlorpromazin hydrochloride, filipin III and L-methyl β-cyclodextrin from Merck (Darmstadt, Germany). All other used chemicals were of analytical grade and received from commercial sources.

### Bacterial strains and bacterial products

*S. pneumoniae* serotype 2 strain D39 wt, D39∆cps and R6x (unencapsulated mutants of D39), R6xΔ*ply* (pneumolysin-deficient mutant of R6x) and R6xΔ*lytA* (autolysin LytA-deficient mutant of R6x) were used and cultured as described previously^27^. For inactivation of bacteria, 50 ml of 10^9^ CFU/ml *S. pneumoniae* culture were heated for 1 h at 56 °C or treated with 70% ethanol on ice. Pneumococci were processed with NaIO_4_ (1 µg/ml), Proteinase K (1000 µg/ml) or Pronase E (100 µg/ml) for 30 min. For the isolation of bacterial, mouse and human DNA pro- and eukaryotic cells were harvested by centrifugation and resuspended in TES, lysed and treated with lysozyme, mutanolysine, RNase, Pronase E and sarcosyl. DNA was extracted and precipitated with phenol, sodium acetate and 2-propanol. Digestion of DNA was performed by overnight incubation of 5 µg isolated DNA with 5 units DNase at 37 °C. RNA isolation was performed using Trizol, chloroform, as well as isopropanol, and for precipitation GlycoBlue was used. Pneumococcal supernatant was generated by incubation of 10^6^ CFU/ml R6x in cell culture medium at 37 °C and in 5% CO_2_ for 24 h. Before stimulation, pneumococci and bacterial components were centrifuged for 10 min at 1800 × *g*. Subsequently, collected supernatants were cultured on Columbia agar plates overnight to confirm bacterial free condition.

### CFU assay

The cells were preincubated with 2 µM CytoD for 30 min before stimulation with 10^6^ CFU/ml R6x. One hour post-infection the cells were treated with 50 µg/ml gentamycin (genta) for one hour. The supernatants (SN) were collected and the cells were lysed with 1% saponin for 10 min. Serial dilutions of the bacterial suspensions were plated on blood agar plates. The colonies were counted after overnight incubation at 37 °C and 5% CO_2_ on blood agar plates and CFUs were calculated.

### Endocytosis and phagocytosis inhibition

Wt BMMs were treated with 2 µM CytoD or 25 nM BafA1 for 30 min to block phagocytosis. Endocytosis was inhibited by treating wt BMMs with 14 µM CP for 30 min, 5 µg/ml Fil III for 1 h or 10 mM MBCD for 30 min.

### KLF4flox/LyzMcre mice

C57BL/6 ERT-Cre^+/−^/Klf4loxP/loxP mice and C57BL/6 ERT-Cre^−/−^/Klf4loxP/loxP mice (Gary K. Owens; Department of Molecular Physiology and Biological Physics, University of Virginia, Charlottesville) and B6.129P2-Lyz2tm1(cre) Ifo mice (Charles River, Sulzfeld, Germany) were mated to generate C57BL/6 LyzMcre^+/+^/Klf4^loxP/loxP^ mice (referred as KLF4^−/−^) and C57BL/6 LyzMCre^−/−^/Klf4^loxP/loxP^ mice (referred as KLF4^+/+^).

### Ethical Statement

Animal housing complied with the Federation of European Laboratory Animal Science Association (FELASA) guidelines and recommendations for the care and use of laboratory animals. The animal procedures were approved by the local institutional (Charité - Universitätsmedizin Berlin) and governmental (Office for Health and Social Affairs Berlin (LAGeSo), approval ID: T0087/15) authorities.

### Cell preparation and cell culture

For the isolation of bone marrow cells, mice were anaesthetized with Ketamine and Xylazine i.p. (both Rotexmedica, Luitré, France) and exsanguinated via the caudal vena cava. BMMs were prepared from femurs and tibiae of wild type- (wt), NOD2^−/−^, TLR2,3,4,7,9^−/−^, TLR9^−/−^, MyD88^−/−^, TRIF^−/−^, STING^−/−^, ASC^−/−^, IFNAR^−/−^, KLF4^+/+^ and KLF4^−/−^ mice that were all on C57BL/6 background. BMMs were cultured in RPMI 1640 medium containing 30% L cell supernatant and 20% FCS and replated 1 day prior to infection in RPMI 1640 containing 15% L cell supernatant and 10% FCS.

### ELISA

Mouse IL-10, IL-6, TNFα and IL-1β liberation in the supernatants were assessed by ELISA Ready-SET-Go! (eBioscience; Frankfurt am Main, Germany) following manufacturer’s instructions.

### Western blots

Cultured cells were stimulated as indicated, washed twice and harvested. For western blot analysis cells were lysed in buffer containing NP40, subjected to SDS-PAGE and blotted on Hybond-ECL membrane (GE Healthcare, Munich, Germany). Membranes were exposed to antibodies specific for mouse KLF4 (GKLF antibody (H-180) rabbit polyclonal IgG, sc-20691), actin, pp38 (all Santa Cruz Biotechnology, Inc., Heidelberg, Germany) overnight at 4 °C and subsequently incubated with secondary antibodies (anti-goat or anti-rabbit either Cy5.5- or IRDye 800–labeled (Rockland, Limerick, PA, USA)) for 1 h at room temperature. Proteins were detected and quantified by using an Odyssey 2.0 infrared imaging system (LI-COR Inc., Bad Homburg, Germany). The quantification was performed with strict regard to the methodical requirements as previously described^49^. The results were either presented as fold of stimulated control (Fig. 5f) or as KLF4 induction percentage of infected control (all other graphs of western blot quantification).

### qPCR

Total RNA was isolated by TRIzol reagent (Thermo Fisher Scientific). 1 µg of total RNA was used to transcribe cDNA (high-capacity cDNA reverse transcription kit; Life Technologies GmbH). After pre-amplification (TaqMan® PreAmp master mix kit) the cDNA was diluted and used for qPCR (TaqMan® gene expression master mix and TaqMan® gene expression assays for GAPDH: Mm99999915_gl and KLF4: Mm00516104_ml; all reagents were obtained from Life Technologies GmbH, Darmstadt, Germany). The protocol was performed according to manufactors instructions. Cycling conditions: 2 min, 50 °C; 10 min, 95 °C and 40 cycles with 15 sec, 95 °C and 1 min, 60 °C.

### Statistics

Statistical analysis was done using Prism 6, GraphPad software (San Diego, U.S.A.). The unpaired T-test was used for Figs 1c, 5f, S1c and S14b and the Mann-Whitney U test for Figs 1b and 7. One way ANOVA with Bonferroni’s multiple comparison test was used when comparing more than two populations.

### Data availability statement

The datasets generated during and/or analysed during the current study are available from the corresponding author upon reasonable request.

## Electronic supplementary material

Dataset 1
