# Supplementary figures and images for: DNA-release by Streptococcus pneumoniae autolysin LytA induced Krueppel-like factor 4 expression in macrophages
Source: Sci Rep. 2018 Apr 10;8:5723. doi: 10.1038/s41598-018-24152-1 (PMC5893607; doi:10.1038/s41598-018-24152-1)

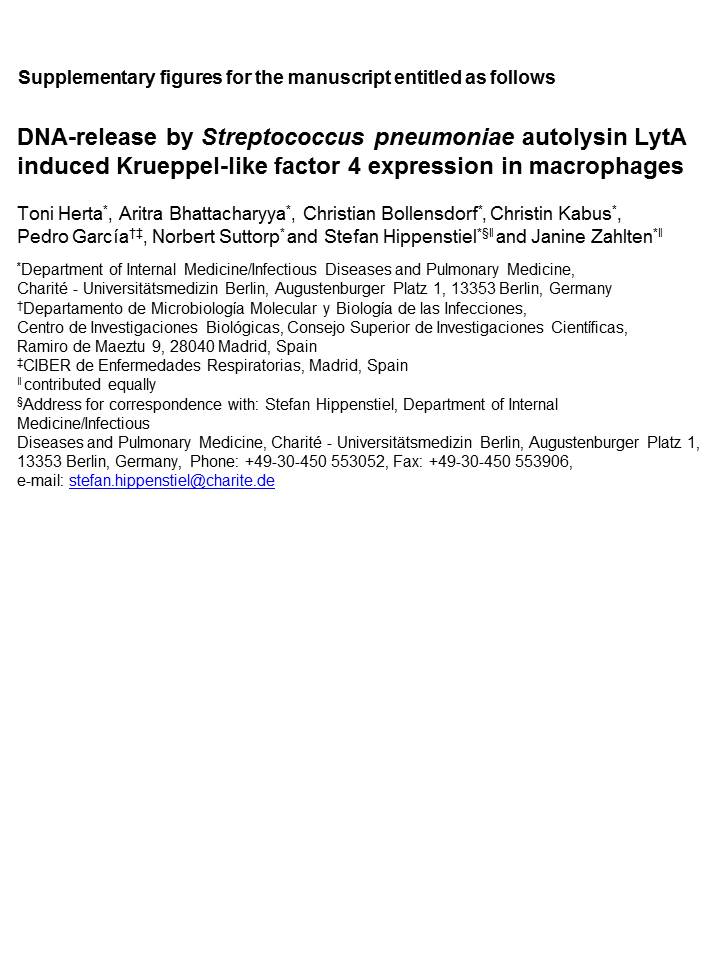


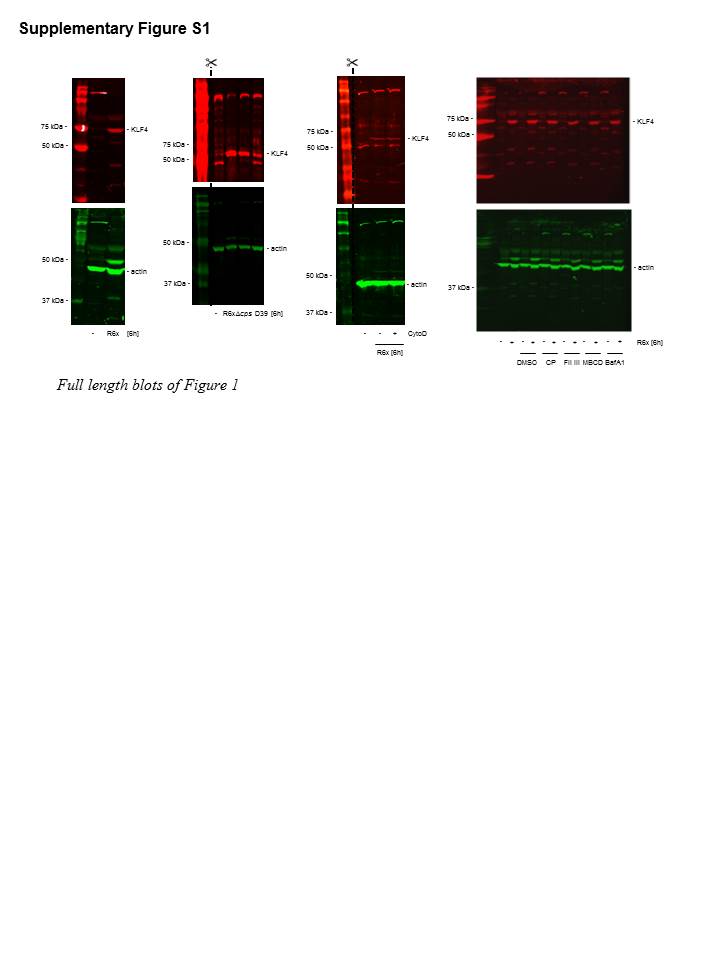


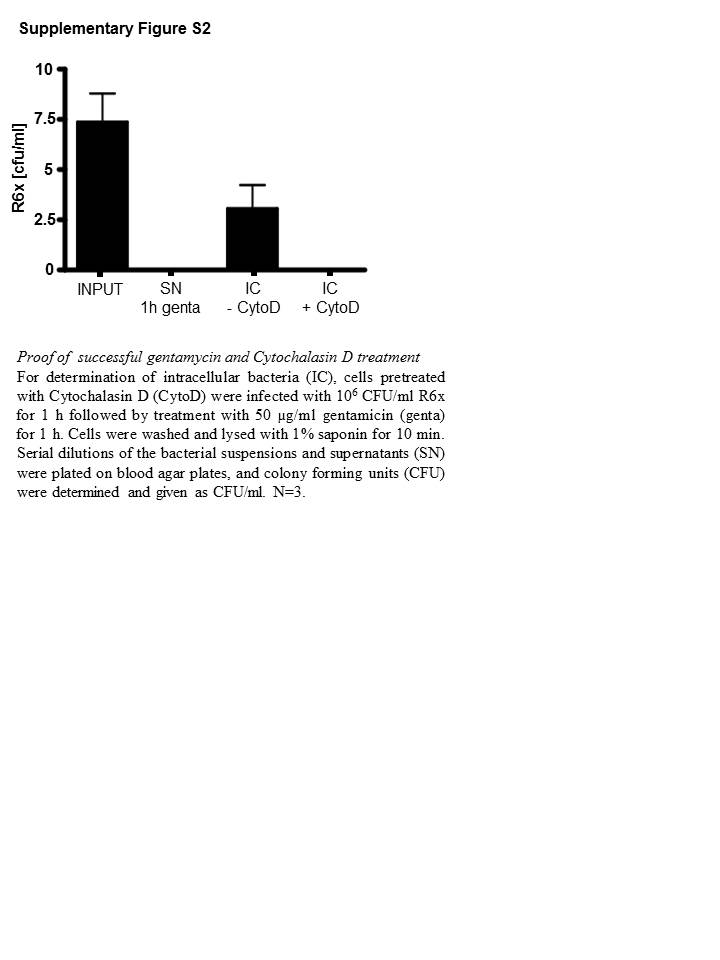


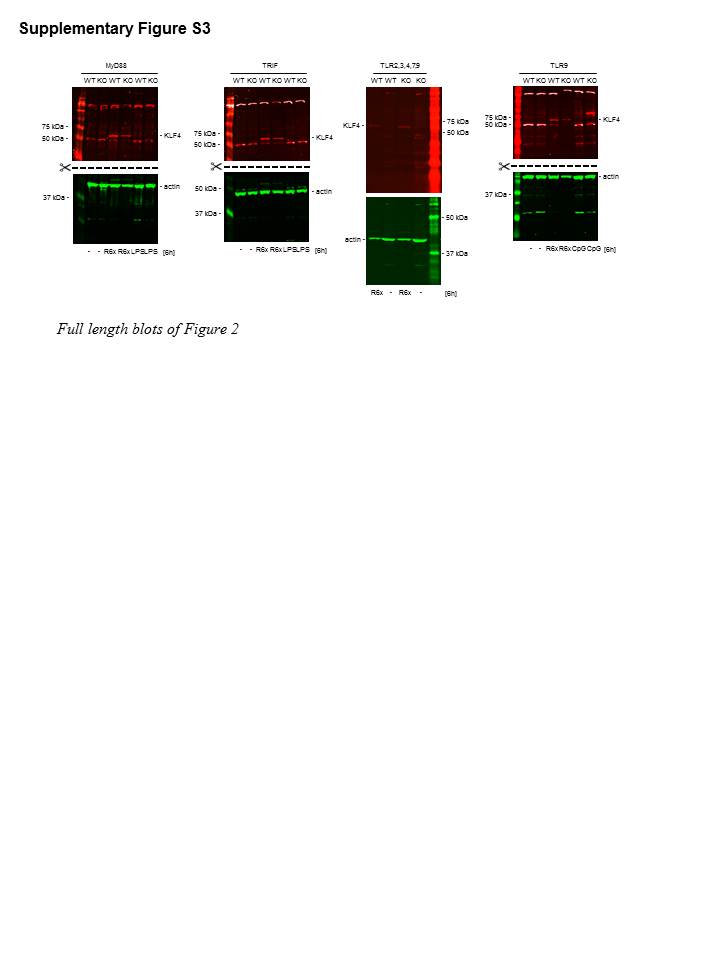


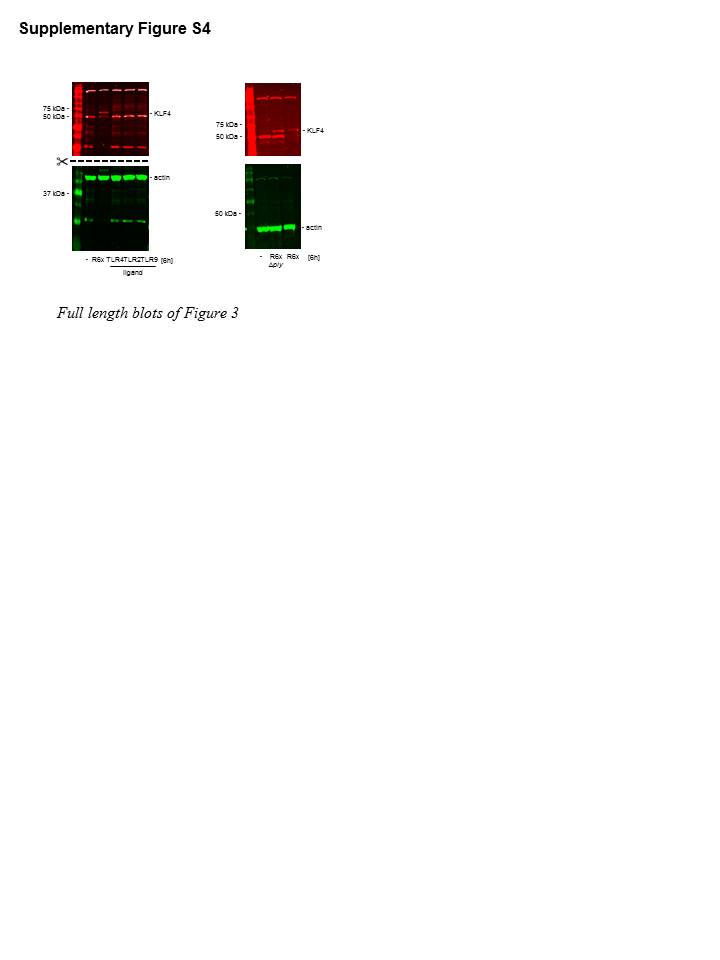


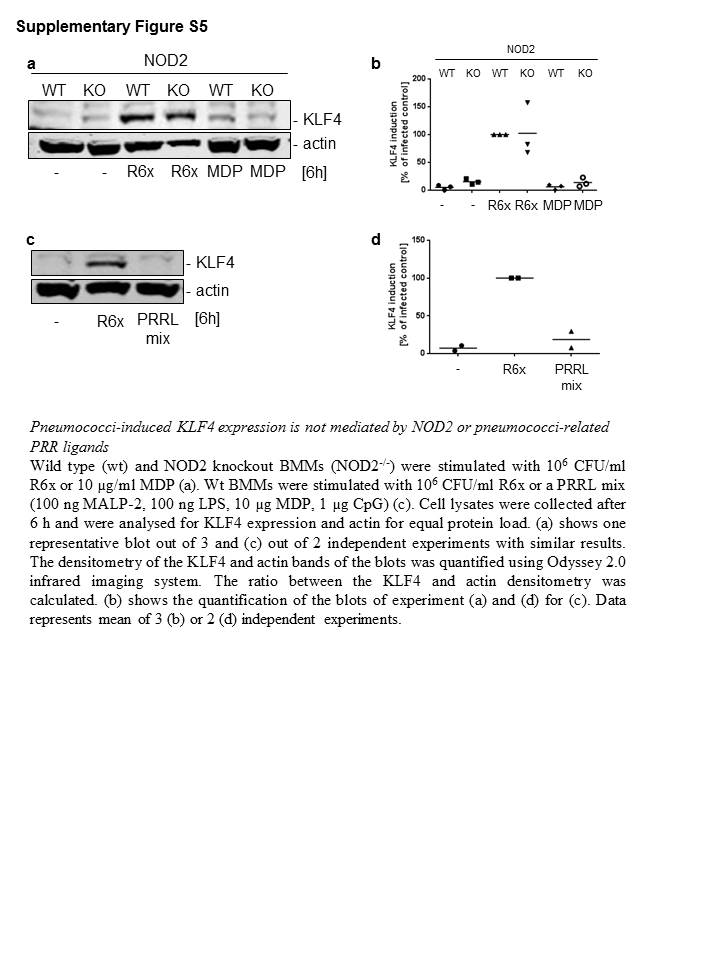


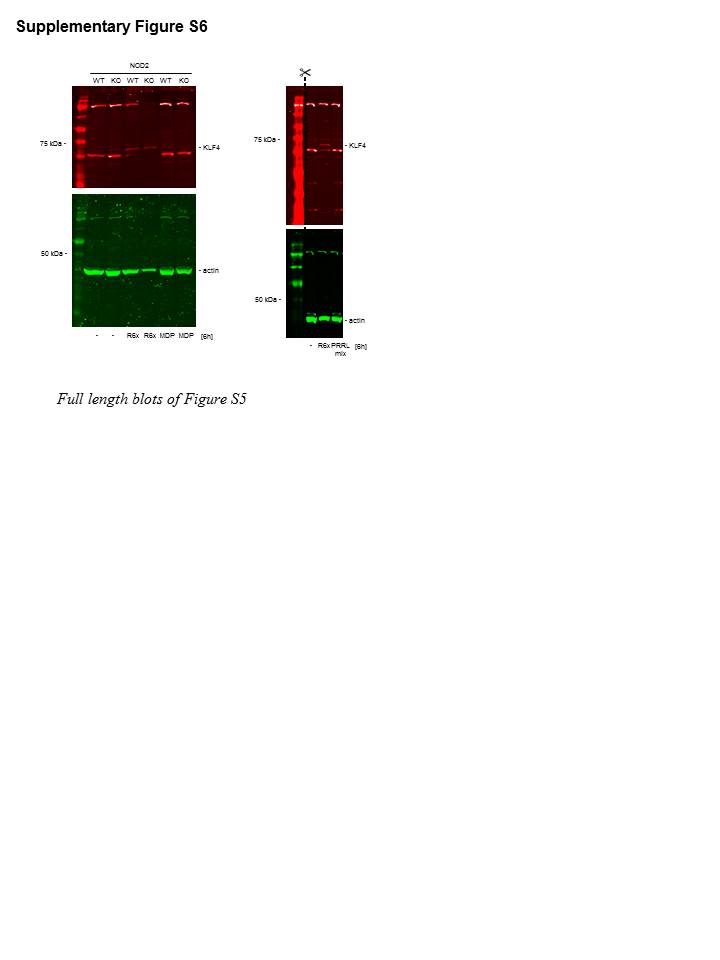


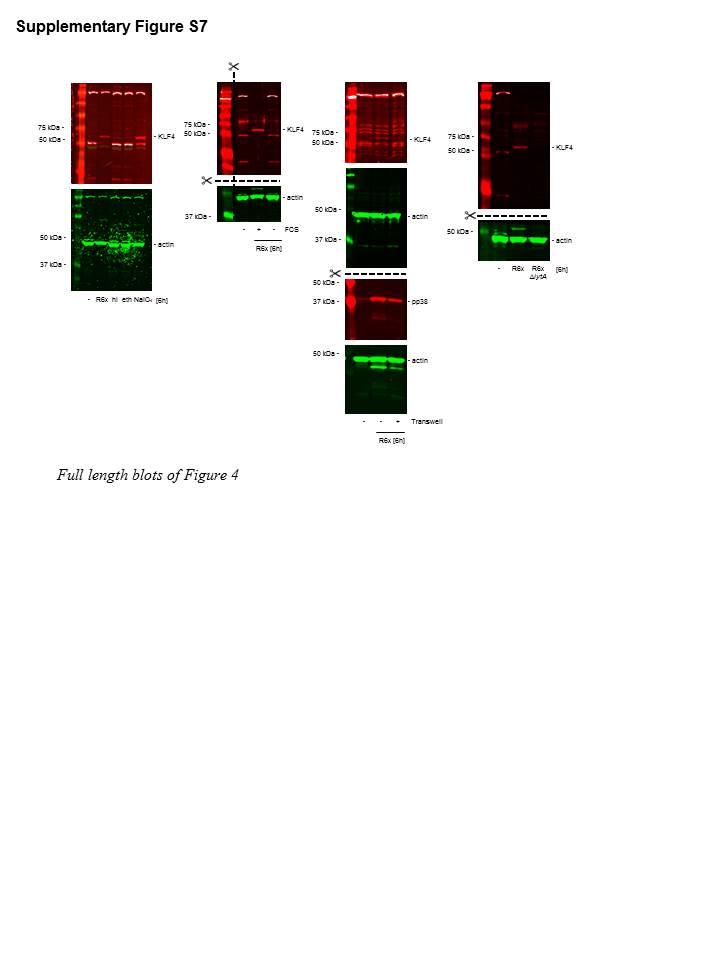


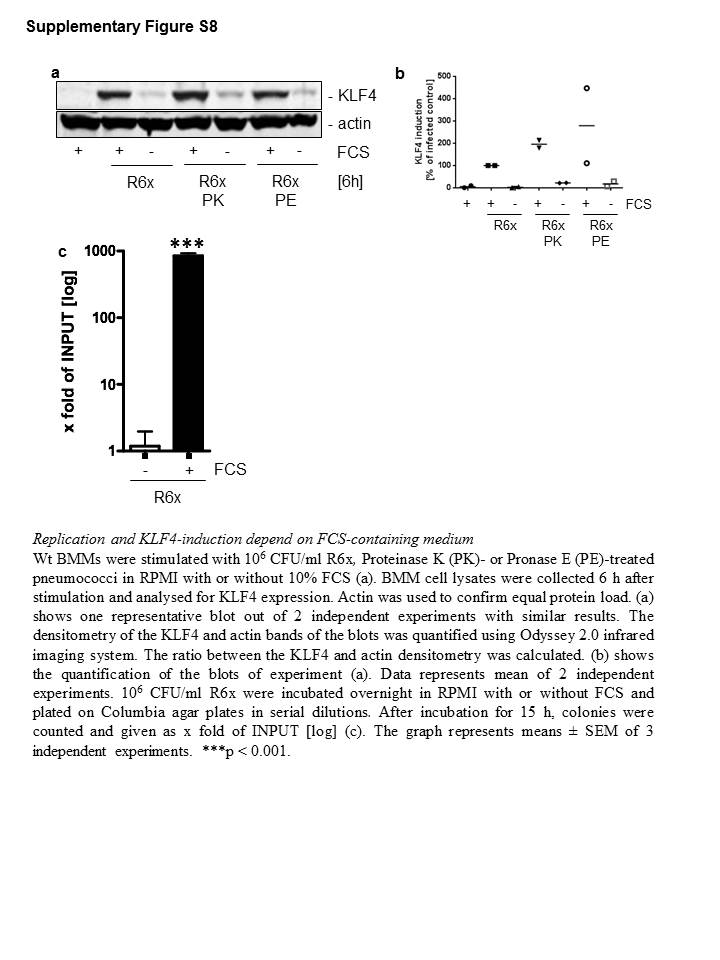


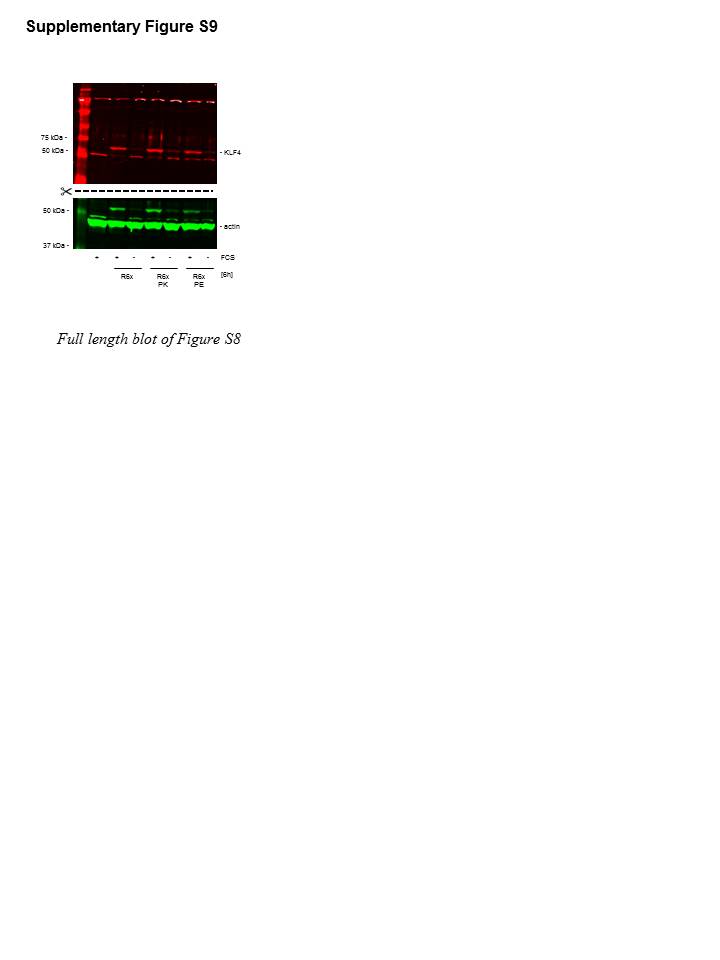


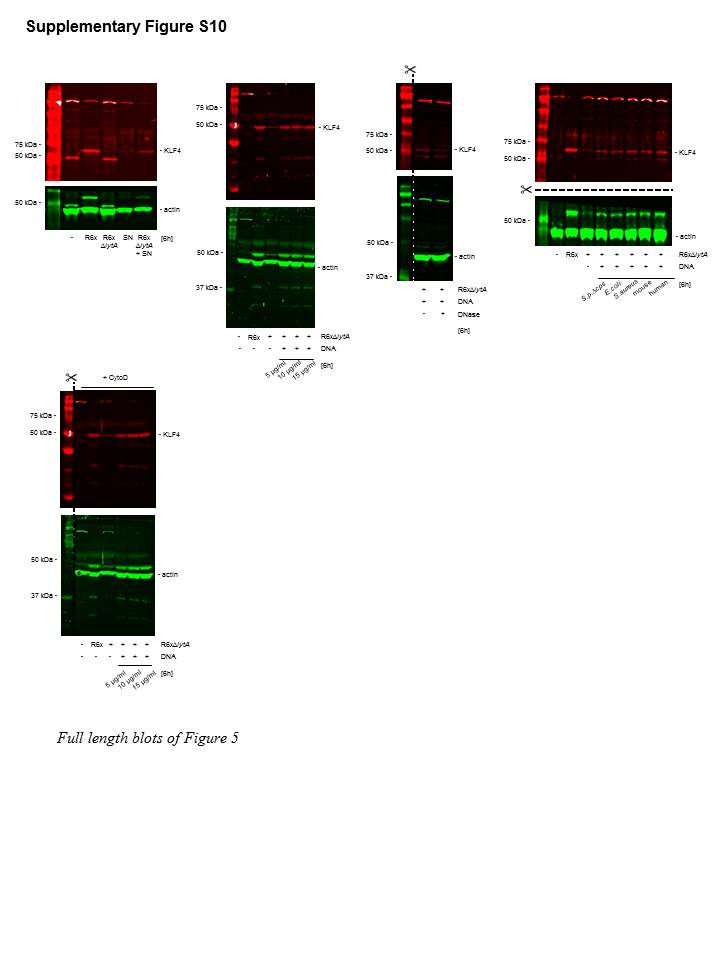


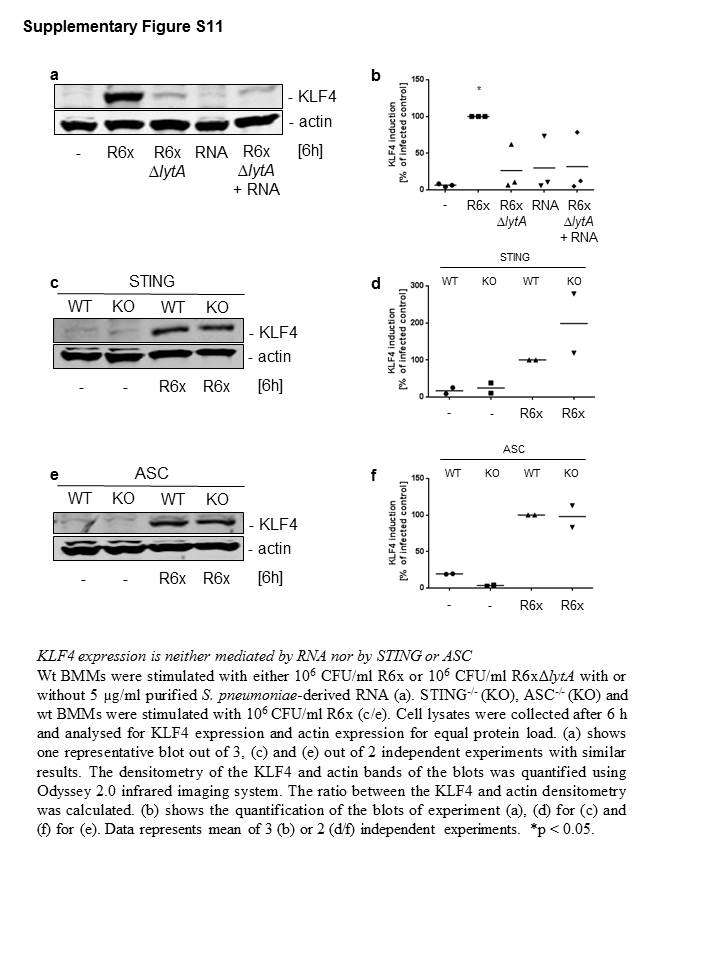


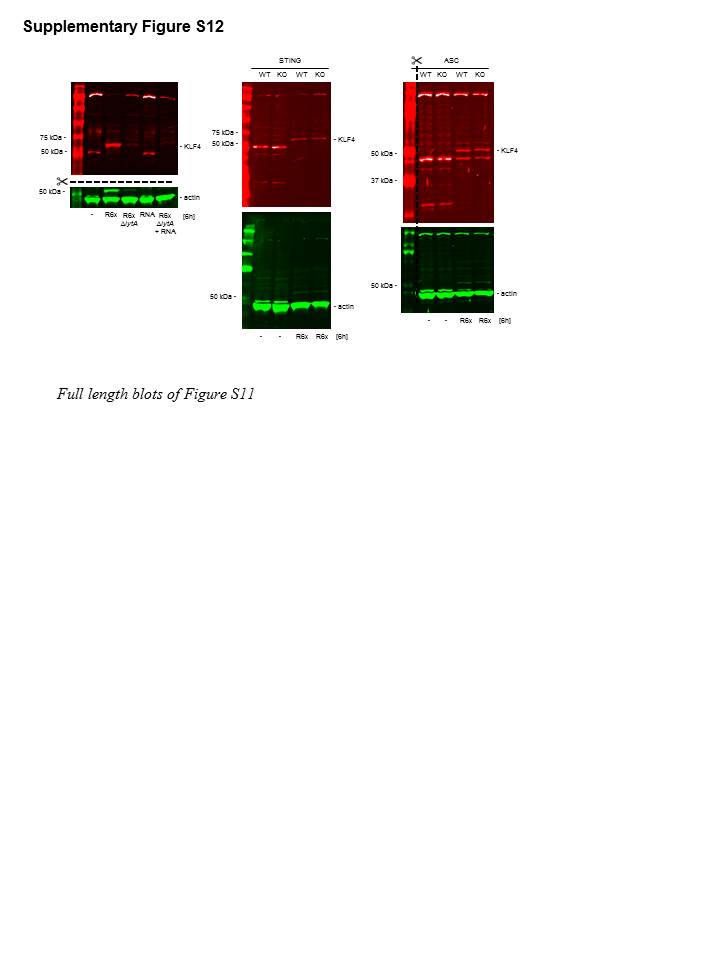


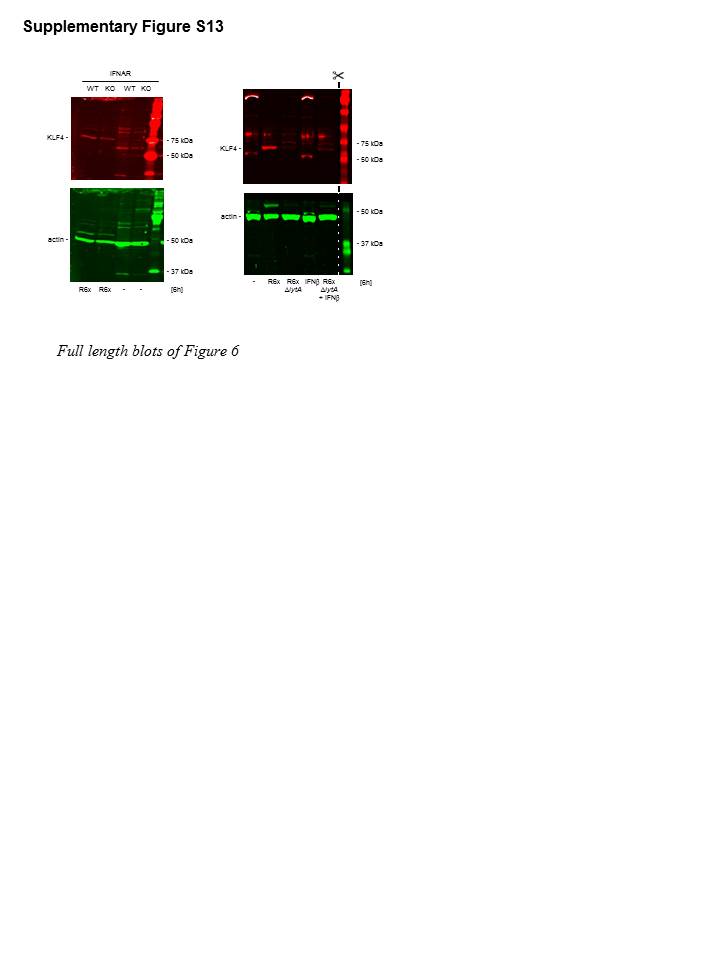


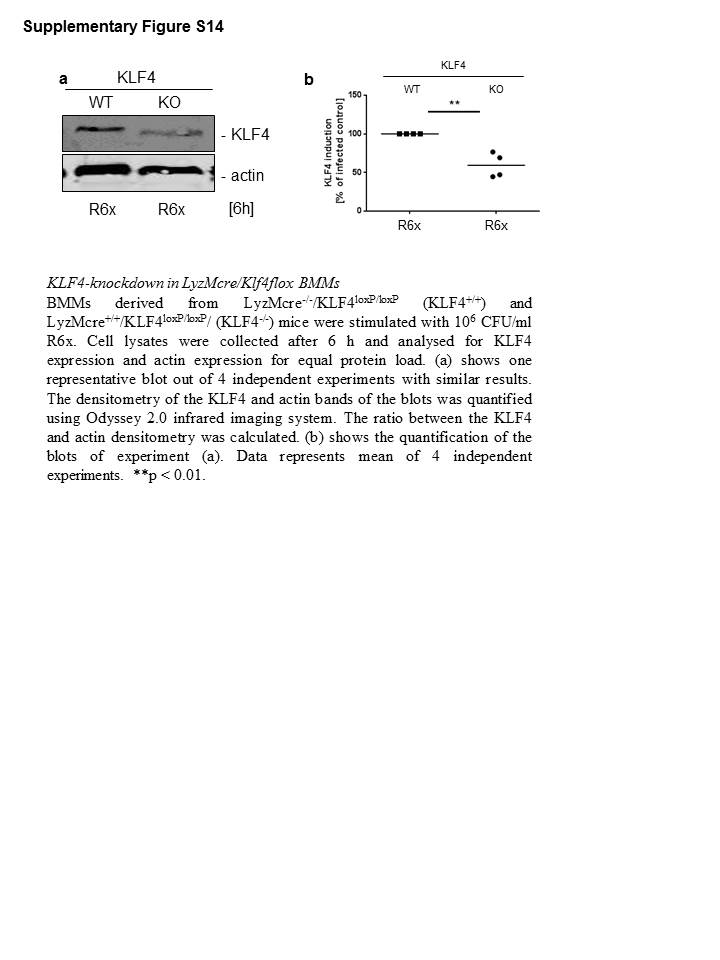


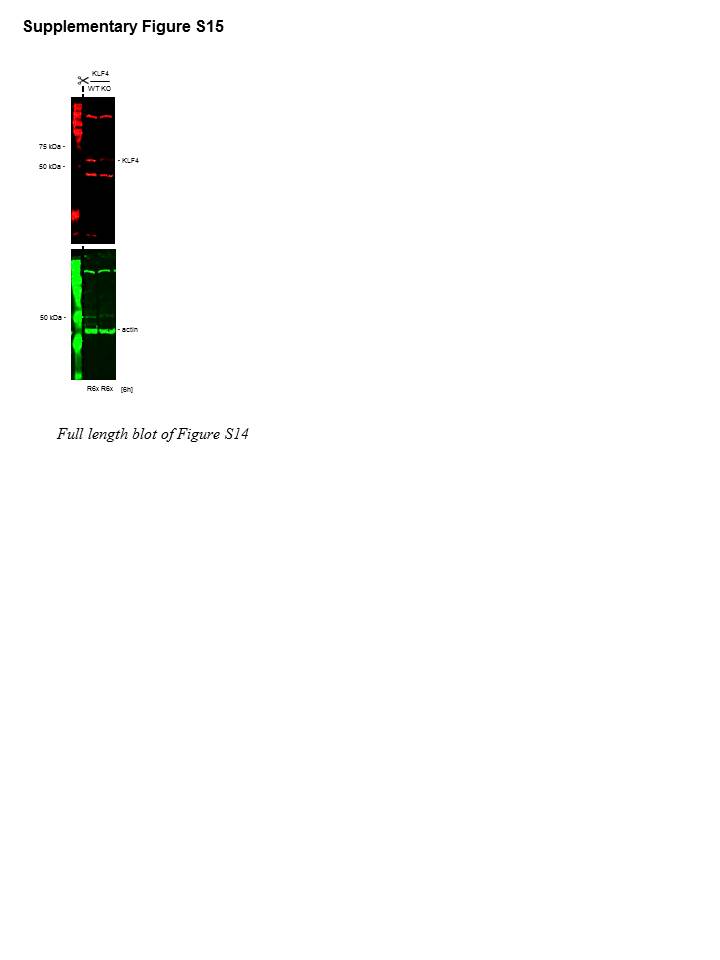

Supplement: Supplementary file 1 — Dataset 1 [file 41598_2018_24152_MOESM1_ESM.doc]
